# The impact of Lacticaseibacillus paracasei GMNL-143 toothpaste on gingivitis and oral microbiota in adults: a randomized, double-blind, crossover, placebo-controlled trial

**DOI:** 10.1186/s12903-024-04251-4

**Published:** 2024-04-20

**Authors:** Min-Kang Lee, I-Hui Chen, I-Ling Hsu, Wan-Hua Tsai, Tzong-Yi Lee, Jhih-Hua Jhong, Bai-Chia Liu, Tsui-Yin Huang, Fang-Kuei Lin, Wen-Wei Chang, Ju-Hui Wu

**Affiliations:** 1 Department of Dentistry, Kaohsiung Medical University Hospital, No.100, Shih-Chuan 1st Road, Sanmin Dist, Kaohsiung City, 807378 Taiwan; 2 Research and Development Department, GenMont Biotech Incorporation, Tainan City, 741014 Taiwan; 3 Institute of Bioinformatics and Systems Biology, National Yang Ming Chiao Tung University, Hsinchu, 300193 Taiwan; 4 Department of Computer Science and Engineering, Yuan Ze University, Taoyuan City, 320315 Taiwan; 5 Departement of Biomedical Sciences, Chung Shan Medical University, No. 110, Section 1, Chien-Kuo N. Rd, Taichung City, 402306 Taiwan; 6 Department of Medical Research, Chung Shan Medical University Hospital, Taichung City, 402306 Taiwan; 7 Department of Oral Hygiene, College of Dental Medicine, Kaohsiung Medical University, Kaohsiung City, 807378 Taiwan

**Keywords:** *Lacticaseibacillus paracasei*, Gingivitis, Probiotic toothpaste, Oral microbiota

## Abstract

**Background:**

This study examines the oral health benefits of heat-killed *Lacticaseibacillus paracasei* GMNL-143, particularly its potential in oral microbiota alterations and gingivitis improvement.

**Methods:**

We assessed GMNL-143’s in vitro interactions with oral pathogens and its ability to prevent pathogen adherence to gingival cells. A randomized, double-blind, crossover clinical trial was performed on gingivitis patients using GMNL-143 toothpaste or placebo for four weeks, followed by a crossover after a washout.

**Results:**

GMNL-143 showed coaggregation with oral pathogens in vitro, linked to its surface layer protein. In patients, GMNL-143 toothpaste lowered the gingival index and reduced *Streptococcus mutans* in crevicular fluid. A positive relationship was found between *Aggregatibacter actinomycetemcomitans* and gingival index changes, and a negative one between *Campylobacter* and gingival index changes in plaque.

**Conclusion:**

GMNL-143 toothpaste may shift oral bacterial composition towards a healthier state, suggesting its potential in managing mild to moderate gingivitis.

**Trial registration:**

ID NCT04190485 (https://clinicaltrials.gov/); 09/12/2019, retrospective registration.

**Supplementary Information:**

The online version contains supplementary material available at 10.1186/s12903-024-04251-4.

## Background

Gingivitis, which is the initial stage of periodontal disease, precedes the more serious stage that is periodontitis. The onset of gingivitis mainly results from the increased number of subgingival microorganisms and alterations in the composition of the subgingival microbiome [1]. Poor oral hygiene can lead to the proliferation of certain Gram-negative anaerobic bacteria, such as *Aggregatibacter actinomycetemcomitans*, *Porphyromonas gingivalis*, *Streptococcus mutans*, and *Tannerella forsythia*. These bacteria play important roles in the initiation and progression of periodontal disease [2–5]. Specific virulence factors, such as lipopolysaccharides and serine-containing lipids, from these Gram-negative oral bacteria can induce periodontal inflammation and potentially trigger gingivitis [6, 7]. However, the mechanism used by the microbiota associated with gingivitis to contribute to the development of periodontitis remains unclear.

A dysbiotic microbial community can be characterized by an enrichment of certain bacterial species; this type of community is associated with periodontal tissue damage [8, 9]. Mouse studies have identified *P. gingivalis* as a key species in the progression of periodontal bone loss [10–12]. This in turn can increase the abundance of the total oral bacteria, which results in a dysbiotic oral microbiota and ultimately triggers an inflammatory response that causes periodontal bone loss. A potential therapeutic approach for such condition can be the reshaping of dysbiotic oral microbiota into a normal symbiotic state, which can potentially alleviate the inflammation and damage caused by periodontal disease.

Probiotics, which confer benefits to human health and provide defense against infectious diseases, are currently being used for microbiota modulation and immune response. Studies explored the preventive and therapeutic potential of probiotics in the context of oral diseases, including periodontal disease, dental caries, halitosis, and *Candida* infection [13, 14]. The potential mechanisms of action of probiotics in maintaining oral health include the following: (1) competition for adhesion sites, colonization sites, and nutrients; (2) production of antimicrobial compounds such as bacteriocins; (3) inhibition of pathogen growth and biofilm formation; (4) direct interaction with pathogens, such as by coaggregation; (5) antagonization of the activity of cytotoxic metabolites produced by pathogens; (6) influencing local (in the oral cavity) and systemic immune responses [15].

In this study, we examined the potential oral health benefits of heat-killed *L. paracasei* GMNL-143 by testing the strain’s ability to coaggregate with oral pathogens and its capacity to inhibit their adhesion to gingival epithelial cells. In addition, we conducted a randomized, double-blind, crossover, and placebo-controlled clinical trial to assess the effect of toothpaste containing the heat-killed GMNL-143 strain on individuals with moderate to severe gingivitis. We also observed the changes in the oral microbiota following the use of GMNL-143 toothpaste.

## Methods

### Bacteria and cells

*Lacticaseibacillus paracasei* GMNL-143 (China Center for Type Culture Collection (CCTCC) number: M2014301; Bioresource Collection and Research Center (BCRC) number: BCRC 910,626) and other *Lactobacillus* strains were obtained from GenMont Biotech Incorporation (Tainan, Taiwan) and cultured on de Man Rogosa and Sharpe (MRS) agar plates (BD Difco™, USA) at 37℃ for 24 h under anaerobic conditions (BD GasPak™ 100 System, BD Bioscience, USA). After washed, *Lactobacillus* strains was resuspended in PBS and then incubated at 121℃ for 15 min to kill bacteria. *S. mutans* ATCC 25,175 and *A. actinomycetemcomitans* ATCC33384 were grown on brain-heart infusion (BHI) agar plates (BD Bacto™, USA). *P. gingivalis* ATCC33277 was grown on BHI agar plates with 5% defibrinated sheep blood (Thermo Fisher Scientific, USA). *Fusobacterium nucleatum* ATCC 25,586 was anaerobically grown on Bruecella agar plates (BD BBL™, USA) with 5% defibrinated sheep blood. *Prevotella intermedia* ATCC 25,611 was grown on tryptic soy agar (TSA; BD Bacto™, USA) plates with 5% defibrinated sheep blood. All oral bacterial pathogens were cultured at 37℃ under anaerobic conditions.

### Removal of SLP from GMNL-143 and preparation of GMNL-143 lysates

Bacteria from overnight culture were harvested by centrifugation at 4000 rpm for 10 min at 4℃ and washed three times with cold sterile deionized water. The bacterial pellet was resuspended in 5 M guanidine hydrochloride (GHCl; Sigma-Aldrich, USA) and incubated at 37℃ with shaking for 60 min to remove non-covalently bound proteins (containing surface layer protein). The GHCl-treated bacteria were collected by centrifugation (4000 rpm, 10 min), washed three times with sterile PBS, and resuspended in PBS for further co-aggregation experiments. To prepare *L. paracasei* GMNL-143 lysates, bacteria were completely homogenized using a bacteriolytic device (FastPrep-24, MP Biomedicals, USA) to break down the bacteria (whole cell lysate), then subjected to high-speed centrifugation (20,000 g, 5 min) at 4℃ to separate the supernatant from the bacterial cell bodies/debris (pellet). Each fraction was applied to the co-aggregation assay.

### Coaggregation assay

Coaggregation of the bacterial mixture was performed according to the protocol described in Methods section of Supporting Information. The absorbance at 600 nm (OD600nm) of the upper layer bacterial mixture was measured using a microplate spectrophotometer (µQuant, BioTek, USA), and the co-aggregation ability was calculated as a report from Keller et al. [16] with a formula of following: OD _*L. paracasei*_ + OD _pathogen_ – OD _(*L. paracasei*+pathogen)_. The higher the value, the better the coaggregation ability of Lactobacillus with oral pathogens.

### Analysis of *L. paracasei* GMNL-143 genome

The *L. paracasei* GMNL-143 isolate was obtained in 2020 from GenMont Biotech Incorporation in Tainan, Taiwan. The whole genome analysis of GMNL-143 was performed by next generation sequencing followed by the annotation of coding genes according to the protocol from a previous report [17].

### Clinical study group/participants

A randomized, double-blind, crossover, and placebo-controlled clinical trial was approved by the Institutional Review Board of Kaohsiung Medical University Chung-Ho Memorial Hospital, Kaohsiung, Taiwan (IRB No. F(I)-20,190,123, registered as NCT04190485 with the first registration date at 09/12/2019 in ClinicalTrials.gov), and was performed in accordance with the relevant guidelines and regulations (from 2020/2/5 to 2022/3/29). The inclusion criteria of subjects were (1) Adults aged between 20 and 59, (2) received a dental prophylaxis one week before enrollment, (3) with ≥ 20 natural teeth, and (4) diagnosed with moderate to server gingivitis. The exclusion criteria included the following: immunodeficiency disease, severe dental caries, mucosal lesions in the oral cavity, current orthodontic treatment, usage of any antibiotics, anti-inflammatory drugs, or probiotic products (excluding yogurt and yogurt drinks) during the trial, pregnancy or lactation, smoking, consumption of alcohol or betel nuts, usage of mouthwash, and participation in another clinical trial. The primary outcome was the gingival index. In this crossover study, Sample size calculation was done considering that gingival index difference between different treatments was 0.4 and standard deviation (SD) was 0.4. After calculated at 0.05 two-sided significance level and power of 90%, a minimum sample size was 24 subjects. As a dropout rate of 10% was considered, approximately 30 subjects were enrolled into this study. Informed consent was obtained from all of the enrolled participants.

### Intervention

One group used probiotic toothpaste (Sodium bicarbonate, glycerol, pure water, Sodium lauryl sulfate, Hydroxy Ethyl Cellulose, Mint spice, Lemon Flavor containing 0.7% heat-killed *L. paracasei* GMNL-143 powder, is about 0.7 × 10^9^ cells/g toothpaste) while the other group used a placebo toothpaste with the same composition indicated above except to GMNL-143 power. The subjects underwent a dental scaling procedure prior to the toothpaste intervention, which made the gingivitis assessment post-scaling being mild-to-moderate gingivitis for all enrolled subjects. After a gingivitis assessment, each patient received 10 min of oral hygiene instruction followed by 30 min of ultrasonic scaling. Subjects brushed their teeth for two minutes twice daily for 4 weeks, followed by a 1-week washout period, and then crossed over to the opposite treatment for another 4 weeks.

### Clinical assessment and sample collections

All subjects received clinical examination of gingival index (GI) [18, 19] and Turesky Modified Quigley-Hein Plaque Index (TMQHPI) [20], collection of samples including subgingival plaque, gingival cervical fluid (GCF) and saliva, halitosis assessment and questionnaires of oral health impact profile (OHIP) and gastrointestinal health at baseline (Visit 1 and Visit 3) and 4 weeks of each phase (Visit 2 and Visit 4). Plaques were collected using sterile periodontal probes and gingival cervical fluid samples were collected by sterile paper points (Sure Dent Corporation, Korea). All collected samples were frozen immediately at -80℃ before further analysis.

### Microbiome evaluation

Bacterial genomic DNA was extracted from the collected plaques and GCF samples using GenElute™ Bacterial Genomic DNA kits (Sigma-Aldrich, USA). A real-time PCR was performed using Rotor-Gene SYBR Green PCR Kit (QIAGEN, Germany) using bacteria-specific primer sets as described in Table S1 and the Methods section of Supporting Information.

### Salivary IgA concentration

IgA concentration in saliva was assessed using Salivary Secretory IgA Indirect Enzyme Immunoassay Kit (Salimetrics, USA) according to the manufacturer’s instruction. Each salivary sample was measured in triplicate. The concentration of sIgA was calculated using the software “Four parameter Logistic Curve” online data analysis tool (MyAssays Ltd., Brighton, Cornwall, UK).

### Statistical analysis

Data were presented as mean ± standard deviation (SD) and statistically analyzed using GraphPad Prism software. For clinical data, continuous variables were analyzed using Dunn’s multiple comparison test or Wilcoxon signed-rank test, and categorical variables were analyzed by Chi-square test. The relationship between relative abundances of oral bacteria and gingival index/plaque index change was analyzed with Spearman’s rank correlation analysis. The in vitro experiments were analyzed using two-tailed t test. Statistical differences were considered significant at *p* value < 0.05.

## Results

### Heat-killed GMNL-143 coaggregates oral bacterial pathogens in vitro

**Fig. 1:**
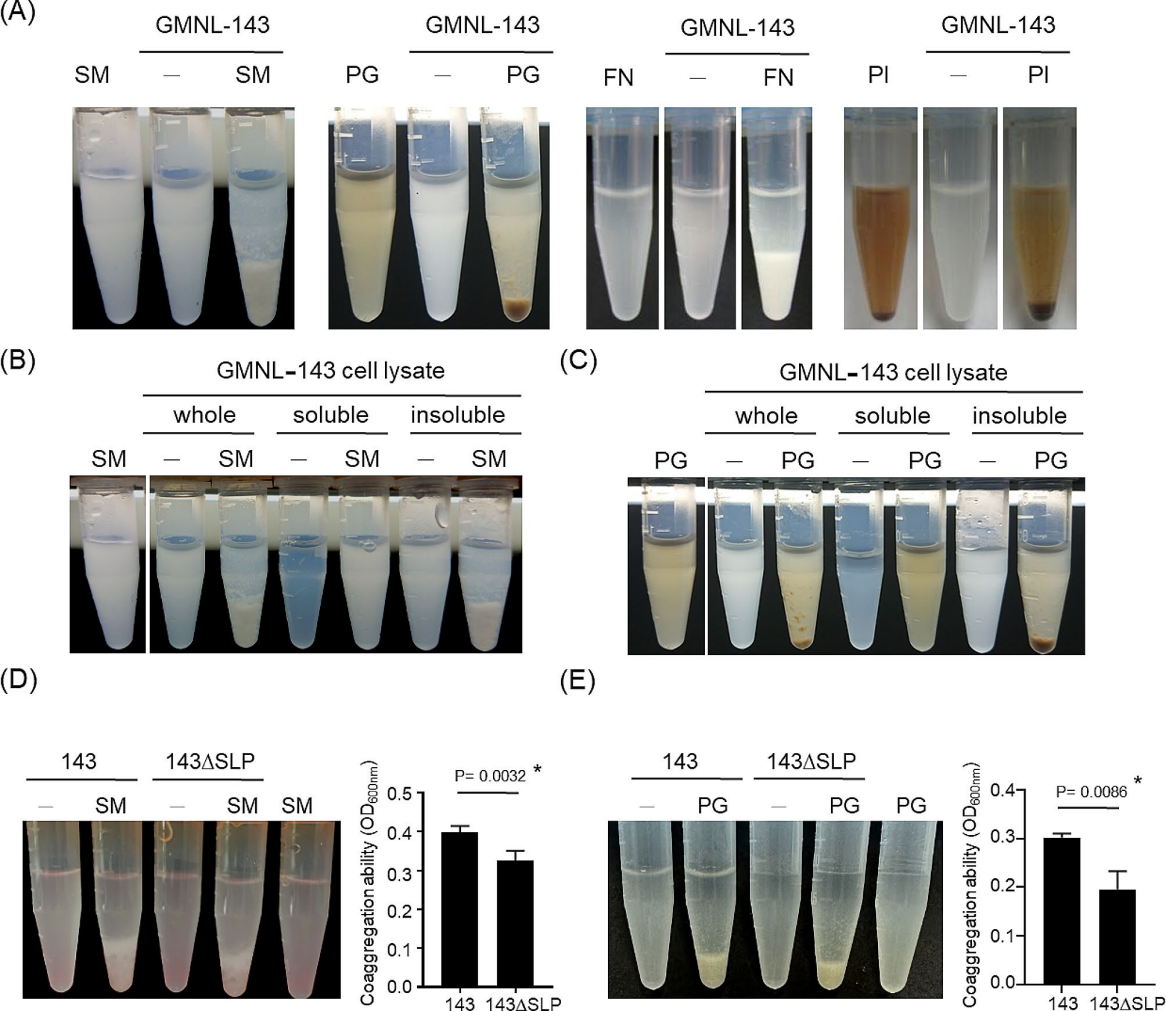
Heat-killed GMNL®-143 displays co-aggregation activity to oral bacterial pathogens. (**A**) the suspensions of oral bacterial pathogens including *Streptococcus mutans* (SM), *Porphyromonas gingivalis* (PG), *Fusobacterium nucleatum* (FN), or *Prevotella. Intermedia* (PI) were prepared as 1 × 10^9^ cells/mL and were added equal cell number of heat-killed GMNL-143 for 30 min at room temperature. (**B, C**) GMNL-143 cell lysate was prepared by adding 1 × 10^9^ cells to 1 ml of lysis buffer, then further divided into soluble or insoluble fractions by centrifugation followed by mixing with SM (BC) or FN (**C**) bacterial suspensions at a volume equivalent to 1 × 10^9^/ml and placed at room temperature for 30 min. (**D, E**) The removal of SLP from GMNL-143 cells were performed by guanidine hydrochloride treatment (143ΔSLP) followed by the co-aggregation assay to SM (**D**) or FN (**E**). Co-aggrgation ability was calculated by the formula of OD _*L. paracasei*_ + OD _pathogen_ – OD _(*L. paracasei*+pathogen)_. Statistical analysis was performed by Student’s *t*-test. *, *p* < 0.05

The coaggregation ability of probiotics to oral bacterial pathogens is one of the selection criteria for the prevention of oral diseases [21]. The addition of heat-killed *Lactobacillus* strains to oral bacterial pathogen culture suspensions of *S. mutans*, *P. gingivalis*, *Fusobacterium nucleatum*, or *P. intermedia* revealed that GMNL-143 was the most effective *Lactobacillus* strain in coaggregating and precipitating all four oral bacterial pathogens (Fig. 1A and Table S2). Heat-killed GMNL-143 was further prepared as a whole-cell lysate and centrifuged into soluble and insoluble fractions, which were then used to test the coaggregation ability. The whole-cell lysate and insoluble fraction, but not the soluble fraction, displayed coaggregation ability with *S. mutans* (Fig. 1B) or *P. gingivalis* (Fig. 1C). *Lactobacillus* surface layer protein (SLP), a hydrophobic protein, promotes bacterial adhesion to intestinal epithelial cells [22]. The coaggregation ability between the original GMNL-143 and the SLP removal counterpart (143∆SLP) was further examined, and a decreased coaggregation ability with *S. mutans* and *P. gingivalis* was observed in their 143∆SLP preparation (Fig. 1D and E, respectively). Similar phenomena were observed in the coaggregation with *P. gingivalis* (Fig. 1D and E). In addition to the coaggregation ability, the preventive effect of heat-killed GMNL-143 on the cell adhesion ability of bacterial pathogens to Smulow-Glickman (SG) oral epithelial cells was determined. After preincubation with heat-killed GMNL-143, *S. mutans* or *P. gingivalis* lost their cell adhesion ability when added to SG cells (Fig. S1). These data suggest that heat-killed GMNL-143 exhibits coaggregation ability with oral bacterial pathogens through SLP, which implies its potential in alleviating dysbiosis-related oral diseases.

**Fig. 2:**
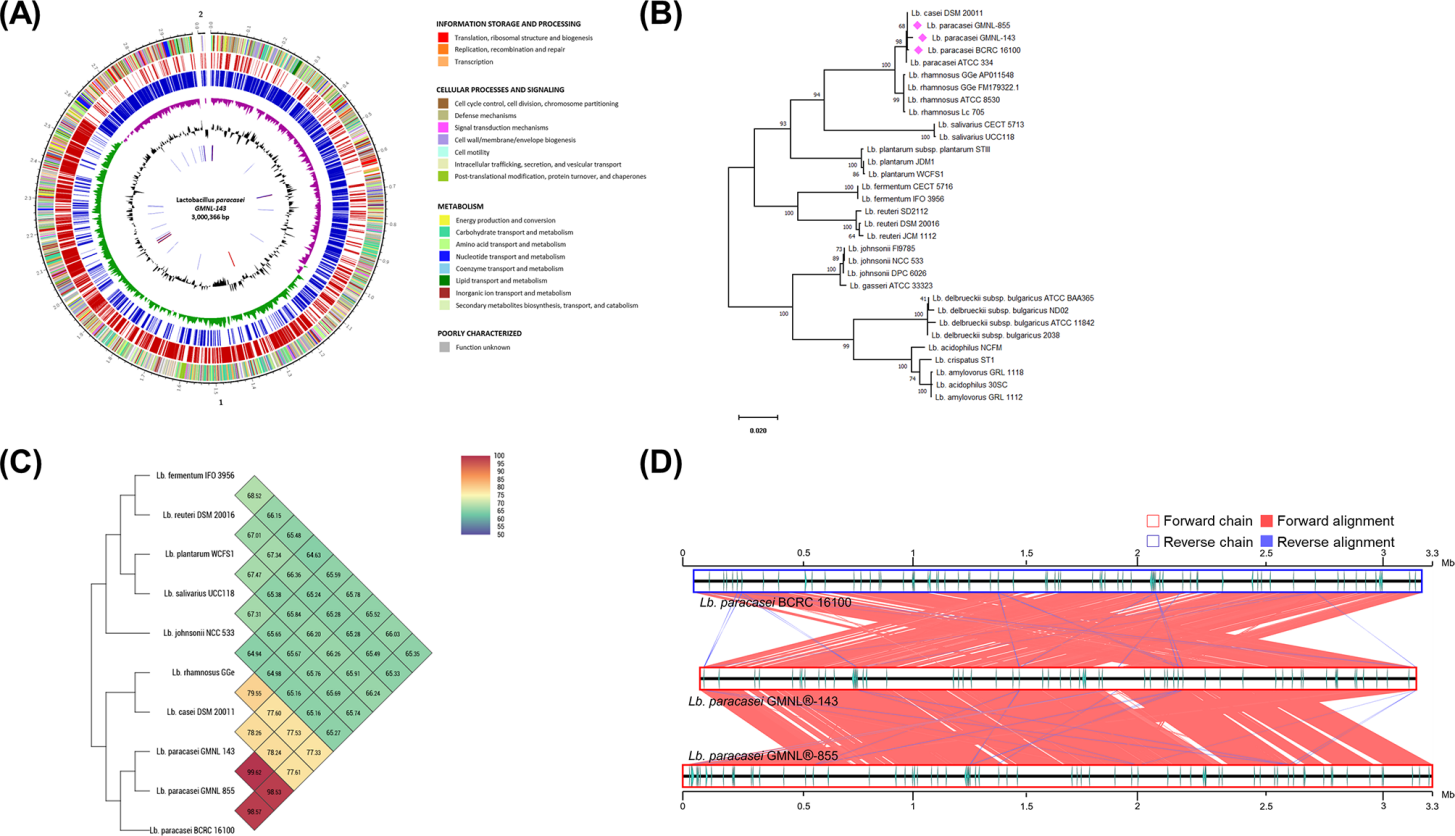
The analysis of whole genome sequencing of GMNL®-143. (**A**) Circular genomic map and KEGG pathway classification of GMNL®-143. (**B**) The neighbor-joining tree of *L. paracasei* GMNL®-143 and other 31 known *Lactobacillus* strains based on the 16 S rRNA gene sequence. (**C**) Heatmap generated with orthologous average nucleotide identity values of GMNL®-143 and the related *Lactobacillus* strains. (**D**) The sequence alignments among BCRC 16,100, GMNL®-143 and GMNL®-855

To explore the unique features of GMNL-143 that render it with potential for improving oral health, we performed whole-genome sequencing by next-generation sequencing and illustrated the genetic organization of GMNL-143 as a circular plot (Fig. 2A). DNA sequencing data analysis revealed 300,366 base pairs of the GMNL-143 genome, which contain 2,867 genes with one prophage-like cluster and three potential bacteriocin genes (Table S3). According to sequence phylogenetic analysis (Fig. 2B), OrthoANI analysis (Fig. 2C), and three strain sequence alignments (Fig. 2D), GMNL-143 is closely related to GMNL-855 and BCRC 16,100; two *L. paracasei* strains were used as controls for the functional analyses of the low coaggregation ability of oral pathogens (Table S2) and anti-inflammatory responses of *L. paracasei* strains [17, 23]. Compared with GMNL-855 or BCRC 16,100, GMNL-143 has several unique genes, including cjaA, capM, epsG, traA, glnP7, and glnPG (Table S4). capM and epsG are involved in bacterial cell wall/membrane/envelope biogenesis and may contribute to the coaggregation ability of heat-killed GMNL-143. After genome annotation, no candidate antibiotic resistance genes nor virulence factor genes have been predicted by CARD and ResFinder in the GMNL-143 DNA chromosome. This finding needs to be further confirmed by polymerase chain reaction (PCR) analysis. In addition, the analysis of the minimum inhibitory concentrations (MICs) of antibiotics against the GMNL-143 strain confirmed that the MICs to GMNL-143 were all below the cutoff value of European Food Safety Authority regulation (Table S5). These data indicate that its use has no safety concern for humans.

### Effects of heat-killed GMNL-143 toothpaste on patients with gingivitis

**Fig. 3:**
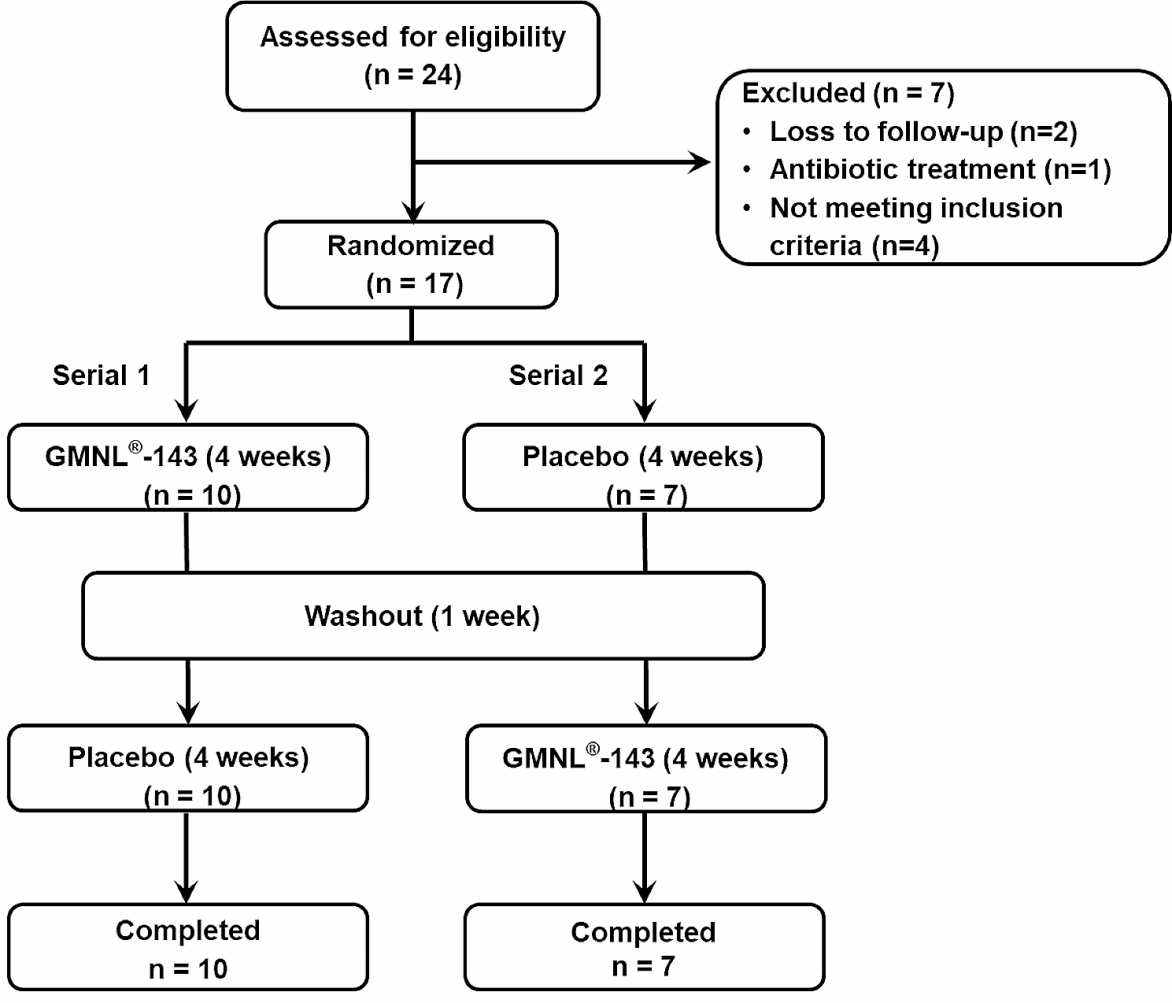
Study design flowchart of the randomized, double-blind, crossover, and placebo-controlled clinical trial. The inclusion and exclusion criteria of enrollment were referred as Methods section

**Fig. 4:**
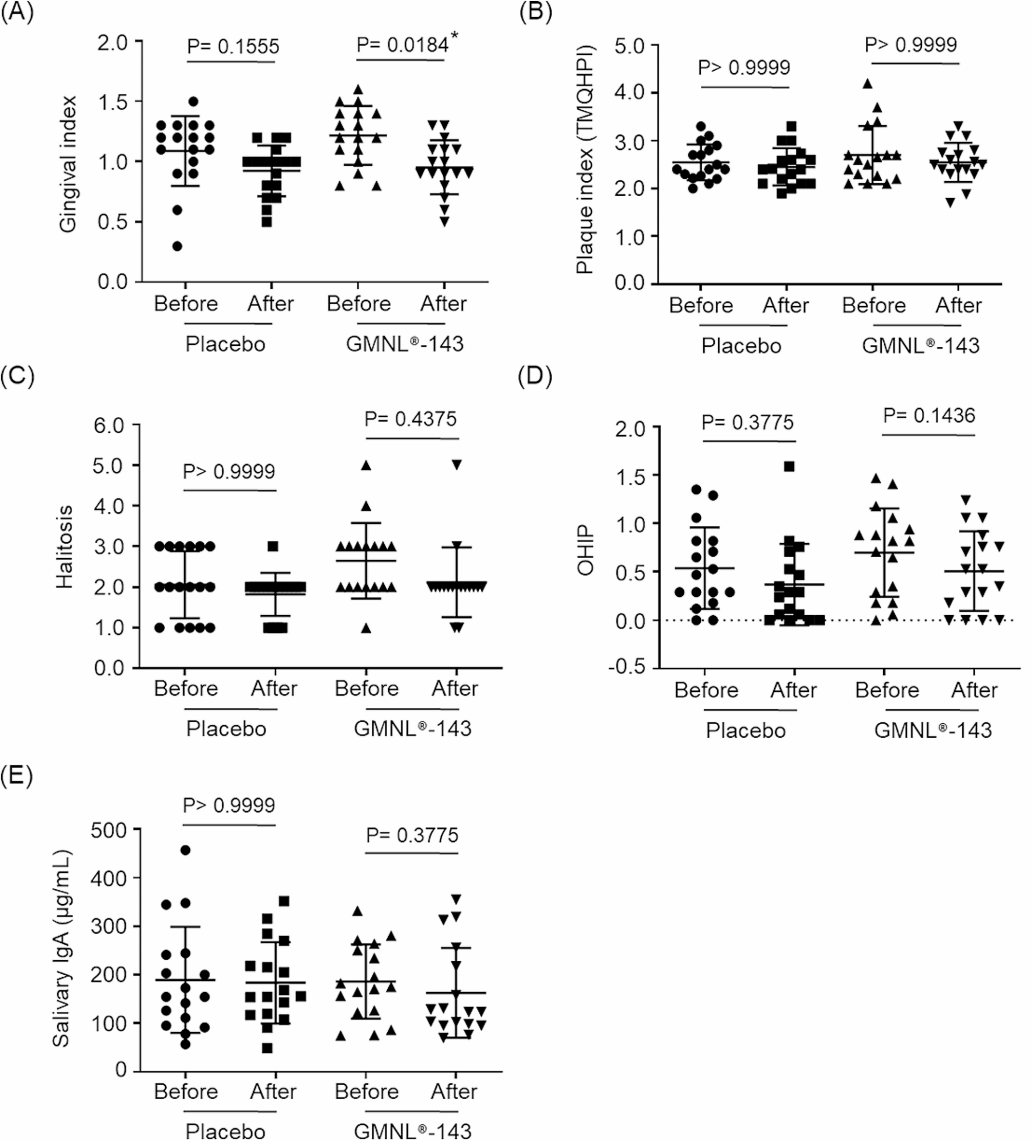
The use of GMNL^®^-143 toothpaste improves gingival index of mild to moderate gingivitis patients. The clinical assessments including gingival index (**A**), plaque index (**B**), halitosis (**C**), OHIP (**D**), or salivary IgA (**E**) of 17 enrolled patients with mild to moderate gingivitis that before and after using control (placebo) or GMNL-143 toothpastes were collected as the descriptions in Methods section. Data were presented as mean ± standard deviation and Dunn’s multiple comparisons test was used for statistical analyses. *, *p* < 0.05

Given its anti-oral bacterial pathogen capabilities, a toothpaste formula containing 0.7 × 10^9^ cells/g heat-killed GMNL-143 was used to evaluate the effect of GMNL-143 on gingivitis in a randomized, double-blind, crossover, and placebo-controlled clinical trial with a setting of 1-week washout period based on previous reports [24, 25]. After confirming the eligibility of the enrolled participants, 17 patients were randomized into two serials of the trial. In Serial 1, the participants used GMNL-143 toothpaste for the first 4 weeks, had a washout period for 1 week, and then used the control toothpaste for the last 4 weeks; in Serial 2, the participants used the control toothpaste for the first 4 weeks, had a washout period for 1 week, and then used the GMNL-143 toothpaste for the last 4 weeks (Fig. 3). The desired subjects in the inclusion criteria were patients with moderate to severe gingivitis, but all the enrolled subjects received a dental scaling procedure before toothpaste intervention, and gingivitis assessment at baseline revealed their mild to moderate gingivitis. The baseline characteristics of the enrolled patients, including age, gingival index, and TMQHPI scores, displayed no difference between the two sequences (Table 1). All data from the control toothpaste (placebo group) and GMNL-143 toothpaste groups were combined for analysis. In this clinical trial, no difference was observed in the amount of toothpaste used by the placebo group versus the GMNL-143 toothpaste group (Fig. S2). The application of GMNL-143 toothpaste decreased the gingival index compared with the baseline (*p* = 0.0184, Fig. 4A), but no statistically significant difference was observed in the plaque index by TMQHPI scoring (Fig. 4B), halitosis (Fig. 4C), OHIP (Fig. 4D), or salivary IgA (Fig. 4E). These data indicate that the application of GMNL-143 toothpaste has beneficial effects on the gingival index of patients with mild to moderate gingivitis.

**Table 1:** Baseline characteristics of enrolled subjects

	All subjects (*n* = 17)			Serial 1 (*n* = 10)			Serial 2 (*n* = 7)			*p*-value^†^
Gender (Male/Female)	9	/	8	6	/	4	3	/	4	
Age	29.82	±	6.82	29.00	±	8.12	31.00	±	4.73	0.3032
Gingival index (mean ± SD)	1.32	±	0.15	1.37	±	0.15	1.26	±	0.13	0.1140
TMQHPI (mean ± SD)	2.83	±	0.60	3.00	±	0.64	2.58	±	0.45	0.1473

^†^Statistical analyses were performed by Mann Whitney test

### Changes in oral microbiota following the application of GMNL-143 toothpaste

**Fig. 5:**
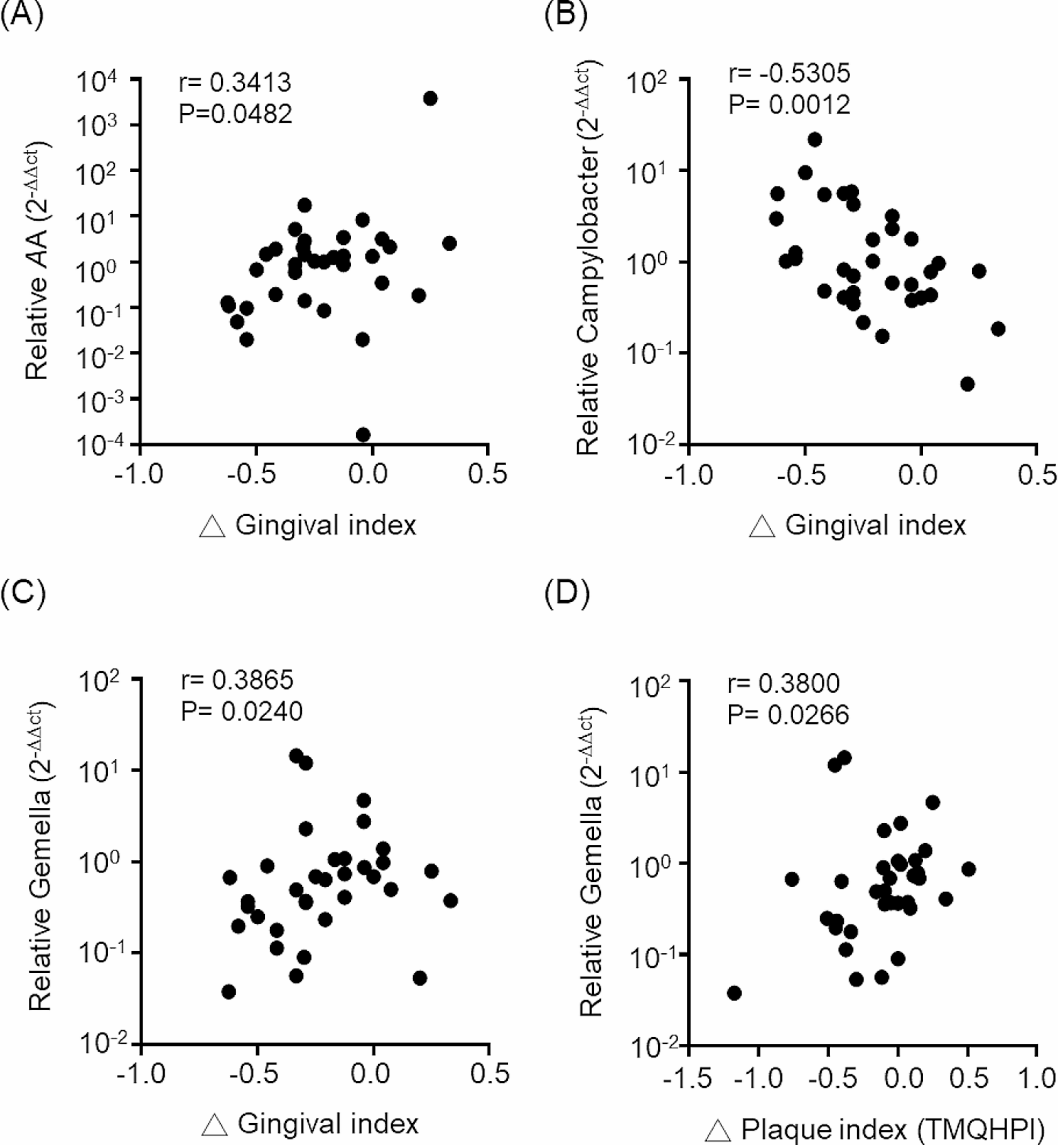
Correlations between oral microbiota in plaque or gingival crevicular fluid specimens and the changes of gingival index or plaque index. The microbiota changes among plaque specimens of all enrolled subjects after using toothpastes were determined by qPCR and were calculated with 2^-ΔΔCt^ method. (**A, B**) The correlations of *A. actinomycetemcomitans* (AA in A) or *Campylobacter* genus (**B**) with the changed gingival index (Δ gingival index) among plaque specimens were determined by with Spearman’s rank correlation analysis. (**C, D**) The correlations of *Gemella* genus with Δ gingival index (**A**) or with the changed plaque index (Δ plaque index) among gingival crevicular fluid specimens were also determined by with Spearman’s rank correlation analysis

We next investigated the effects of GMNL-143 toothpaste on oral microbiota by extracting the total DNA from plaque or the gingival crevicular fluid (GCF) and subsequently conducting quantitative PCR (qPCR) using specific primers for several oral bacteria. The microbiota analysis revealed a decreased trend in most oral pathogenic bacteria after the use of GMNL-143 toothpaste, but only the decrease in S. mutans in the GCF samples was notable. However, this result did not reach statistical significance (from 1.52 ± 1.82 to 0.61 ± 0.63, *p* = 0.0655, Table 2). *Campylobacter*, a genus of bacteria usually found in the healthy oral cavity, showed an increase in the plaque samples after the use GMNL-143 toothpaste, but this change was not significant (from 1.29 ± 1.73 to 2.41 ± 2.59, *p* = 0.1754, Table 2). Pooling all the qPCR data revealed a significant positive correlation between *A. actinomycetemcomitans* and changes in the gingival index (Fig. 5A) and a negative correlation between *Campylobacter* and changes in the gingival index (Fig. 5B) in the plaque samples. As for the GCF samples, positive correlations were observed between *Gemella* and changes in the gingival (Fig. 5C) and plaque indexes (Fig. 5D). These results suggest that the use of toothpaste containing GMNL-143 among patients with mild to moderate gingivitis can modify the composition of their oral microbiota to closely resemble that of a normal, healthy individual.

**Table 2:** Bacterial composition of plaques and gingival crevicular fluid (GCF) from gingivitis patients.^†^

	Plaque			GCF		
	Placebo	GMNL-143	*p*-value^‡^	Placebo	GMNL-143	*p*-value^‡^
**2** ^**-ΔΔCT**^	**Mean ± SD**	**Mean ± SD**		**Mean ± SD**	**Mean ± SD**	
Total bacteria	0.95 ± 0.53	0.87 ± 0.34	0.8536	1.48 ± 1.64	1.08 ± 0.84	0.6441
*Porphyromonas*	5.83 ± 13.11	3.30 ± 8.52	0.4143	1.78 ± 2.34	1.75 ± 1.90	0.3028
*Treponema*	1.39 ± 1.70	2.31 ± 1.81	0.1167	1.72 ± 2.13	2.99 ± 3.60	0.1876
*Tannerella*	3.13 ± 4.93	1.16 ± 0.94	0.5879	1.59 ± 1.66	1.69 ± 1.45	0.3757
*Filifactor*	1.20 ± 0.46	1.37 ± 0.92	> 0.9999	1.73 ± 1.34	1.52 ± 0.99	0.9632
*Aggregatibacter*	2.24 ± 3.71	1.73 ± 3.06	0.7467	1.82 ± 2.68	1.53 ± 1.19	0.7119
*Streptococcus mutans*	1.34 ± 1.97	1.96 ± 2.22	0.1102	1.52v± 1.82	0.61 ± 0.63	0.0655
*Porphyromonas gingivalis*	1.22 ± 1.36	0.89 ± 0.81	0.7090	0.64 ± 0.47	1.03 ± 0.79	0.0984
*Fusobacterium nucleatum*	0.97 ± 0.56	1.21 ± 0.59	0.1270	ND	ND	ND
*Aggregatibacter actinomycetemcomitans*	1.58 ± 1.37	1.35 ± 2.11	0.2297	ND	ND	ND
*Streptococcus*	1.30 ± 0.88	0.87 ± 0.77	0.3755	1.08 ± 0.76	0.87 ± 0.63	0.1202
*Haemophilus*	1.61 ± 2.00	1.60 ± 2.05	0.8900	1.32 ± 1.46	1.33 ± 1.08	0.7820
*Capnocytophaga*	1.54 ± 1.53	1.49 ± 1.43	0.5416	1.65 ± 2.08	1.79 ± 1.51	0.6441
*Gemella*	1.31 ± 1.07	1.03 ± 0.73	0.3755	0.64 ± 0.55	0.90 ± 1.21	0.3028
*Campylobacter*	1.29 ± 1.73	2.41 ± 2.59	0.1754	1.44 ± 1.26	2.39 ± 2.09	0.1591
*Granulicatella*	0.69 ± 0.70	0.93 ± 0.91	0.7057	0.70 ± 0.66	1.36 ± 2.01	0.5171

^**†**^Bacterial composition was determined by the qPCR method and calculating the value of 2^−ΔΔCt^ that reflected the fold change compared to the baseline of bacterial contents from plaque or GCF specimens

^‡^Statistical analyses between GMNL®-143 and placebo toothpaste were performed by Wilcoxon signed-rank tes

## Discussion

The removal of SLPs from GMNL-143 reduced its coaggregation function with *S. mutans* or *P. gingivalis* (Fig. 1D and E, respectively). Meng et al. demonstrated the coaggregation ability of SLPs from several *Lactobacillus* strains to *Escherichia coli* or *Salmonella typhimurium* [26]. GMNL-143 also displayed prevention ability against biofilm formation by *S. mutans*. The addition of GMNL-143-derived lipoteichoic acid (LTA) or peptidoglycan (PGN) reduced *S. mutans* biofilm formation in a dose-dependent manner (Fig. S3). Ahn et al. found that L. plantarum-derived LTA, but not PGN, inhibited the biofilm formation of *S. mutans* through D-alanine moieties on LTA [27]. LTA and PGN have been considered the main bioactive components in postbiotics based on their immunomodulatory activity. The LTA of *L. plantarum* can reduce Pam2CSK4-induced interleukin-8 production in human intestinal epithelial cells [28]. PGN and the derived muropeptides of *L. salivarius* Ls33 displayed anti-inflammatory activity in a 2,4,6-trinitrobenzenesulfonic acid-induced colitis mouse model through the induction of CD103 + regulatory dendritic cells [29]. In our clinical trial, the use of GMNL-143 toothpaste reduced the gingivitis index (Fig. 2A), which suggests the anti-inflammatory activity of GMNL-143. The anti-inflammatory capability of LTA or PGN of GMNL-143 can be investigated in the future.

Chen et al. reported that heat-killed probiotic strains, including several *Lactobacillus* strains or *Bifidobacterium animalis* subsp. *lactis* AP-32, displayed direct antibacterial activities against oral pathogens, including *S. mutans*, *P. gingivalis*, and *F. nucleatum* [30]. Using an inhibition zone assay, we examined the antibacterial activity of GMNL-143 in live and heat-killed formats and observed that both can suppress the growth of *P. gingivalis* or *F. nucleatum* but not *S. mutans* (Table. S6). However, the antibacterial activity of GMNL-143 was abolished at neutral pH (Table S6). In addition, no evident growth inhibition was observed when the heat-killed GMNL-143 powder was used in the GMNL-143 toothpaste in the inhibition zone experiments (Table S6). These data suggest that the antibacterial activity of GMNL-143 is associated with acidic pH. The beneficial effects of GMNL-143 toothpaste on mild to moderate gingivitis are likely to be associated with the coaggregation of oral bacterial pathogens, prevention of the adherence of oral bacterial pathogens to oral epithelium, and inhibition of biofilm formation. This also suggests that maintaining the acidic pH of GMNL-143 toothpaste may improve the suppression of the growth of oral bacterial pathogens.

The results showed that GMNL-143 toothpaste can alter the composition of the oral microbiota to closely resemble that of normal healthy individuals, although no statistically significant differences were achieved (Table 2), possibly due to the small sample size and short usage time. In the analysis of data from the two serials within this trial separately, significant reductions were observed in the gingival index after the use of GMNL-143 toothpaste in both serials (Fig. S4A). Although no statistically significant difference was detected in the salivary IgA concentration in the combined analysis (Fig. 4E), a significant decrease in salivary IgA was observed in Serial 1 after four weeks of using GMNL-143 toothpaste (Fig. S4B). The relationship between chronic periodontitis and salivary sIgA levels has been investigated in several studies. In smokers with periodontitis patients, sIgA concentrations were lower compared to non-smokers [31]. However, non-surgical periodontal treatment with scaling and root planing was associated with reduced levels of sIgA [32]. Gingival inflammation was associated with significantly higher levels of sIgA, particularly in children with spastic cerebral palsy (CP) who lack cervical motor control [33]. In addition, a study investigating the beneficial effects of probiotics on oral mucosal immunity found increased sIgA levels after 6 weeks of consumption [34]. These findings suggest that there is a complex relationship between sIgA levels and gingival inflammation, and that factors such as smoking, periodontal treatment, and specific health conditions such as CP may influence gingival inflammation. *Tannerella*, which belongs to the “red-complex” gram-negative bacteria that proliferate in periodontal disease [35], showed a significant reduction after GMNL-143 toothpaste had been used in the plaque samples of Serial 1 group (*p* = 0.0164, Table S7) or GCF samples of Serial 2 (*p* = 0.0175, Table S8) compared with the placebo. In a mouse model, the infection of *T. forsythia* resulted in increased alveolar bone loss compared with the noninfected control [36]. The abundance of total *Campylobacter* genus increased in plaque samples with decreased gingival index after using GMNL-143 toothpaste (Fig. 5B). Thus, changes in the abundance of a particular *Campylobacter* may reflect the progression of periodontitis. Henne et al. demonstrated the decreased prevalence of *C. concisus* and *C. curvus* in the subgingival plaque of periodontitis patients [37]. Our results revealed that the abundance of the total *Gemella* decreased in the GCF samples with reduced gingival (Fig. 5C) and plaque indexes (Fig. 5D). However, Miyoshi et al. observed a decrease in *G. haemolysans* in the salivary samples of periodontitis patients compared with those from healthy controls; thus, *G. haemolysans* displayed growth inhibition ability against *P. gingivalis* [38]. It indicates that investigating the changes in specific bacterial species, such as *C. concisus, C. curvus*, or *G. haemolysans* may be required to further gain insights into the effect of probiotic interventions, such as GMNL-143 toothpaste used in this study, on the changes in oral microbiota.

## Conclusions

Gingivitis, driven by imbalances in oral microbiota, can progress to severe periodontal disease. Probiotics, especially *L. paracasei* GMNL-143, offer a promising approach to adjust oral microbiota and alleviate gingivitis. In our study, heat-killed GMNL-143 displayed the capability to coaggregate with oral pathogens and inhibit their attachment to gingival cells. Our double-blind clinical trial further highlighted the efficacy of GMNL-143 toothpaste in decreasing gingival indices and reducing *S. mutans* concentrations in gingivitis patients. These findings emphasize the potential of GMNL-143 toothpaste as a novel probiotic-based strategy for managing gingivitis, promoting overall oral health, and averting its evolution into advanced periodontal conditions.

### Electronic supplementary material

Below is the link to the electronic supplementary material.

Supplementary Material 1

## Data Availability

Whole genome sequences of GMNL-143, GMNL-855, and BCRC 16100 have been deposited in BioProject (https://www.ncbi.nlm.nih.gov/bioproject/) with the accession numbers of PRJNA773974 (GMNL-855 and BCRC 16100) and PRJNA915772 (GMNL-143).

## Abbreviations

GCF: gingival cervical fluid
LTA: lipoteichoic acid
MICs: minimum inhibitory concentrations
OHIP: oral health impact profile
PGN: peptidoglycan
SLP: surface layer protein
TMQHPI: Turesky Modified Quigley-Hein Plaque Index
