# Supplementary material for: The impact of Lacticaseibacillus paracasei GMNL-143 toothpaste on gingivitis and oral microbiota in adults: a randomized, double-blind, crossover, placebo-controlled trial
Source: BMC Oral Health. 2024 Apr 20;24:477. doi: 10.1186/s12903-024-04251-4 (PMC11031891; doi:10.1186/s12903-024-04251-4)
Supplement: Supplementary file 1 — Supplementary Material 1 [file 12903_2024_4251_MOESM1_ESM.pdf]

Supporting information

I. Supporting Materials and Methods

Cell culture

Smulow-Glickman (SG) human gingival epithelial cells were cultured with Dulbecco's modified Eagle's medium (DMEM; Thermo Fisher Scientific, USA) supplemented with 10% heat-inactivated fetal bovine serum (Atlanta Biologicals, USA).

Oral epithelial cell adhesion assay

2×10^5 SG human gingival epithelial cells were seeded onto glass cover slips which were placed in a 24-well plate and incubated at 37°C for 24 hours. *L. paracasei* GMNL®-143 and pathogenic bacteria (2×10^9 cells/mL) were subjected to co-aggregation assay. After 10 minute of coaggregation, 100 uL of upper layer bacterial mixture was collected, mixed with 400 uL serum and antibiotic-free DMEM medium, and then added to SG cells and incubated at 37°C for 1 hour. SG cells were washed 3 times with PBS, fixed with 2.5% glutaraldehyde for 30 minutes, and stained with Gram staining for microscopic examination (BX51, Olympus C&S, Japan).

Extraction of LTA and PGN from *Lactobacillus*

For LTA purification, heat-treated *L. paracasei* GMNL®-143 was dissolved in 0.05 M sodium acetate (pH= 4.0) and lyzed with an ultrasonic homogenizer (model W-220F, Heat Systems-UltraSonic, Inc., USA). After centrifugation, the bacterial pellet was resuspended in chloroform:methanol:water (1:1:0.9, v/v) and the aqueous phase containing LTA was collected. After evaporation of residual methanol, LTA was applied to a column (2.5×20 cm) of Octyl-Sepharose CL-4B (Sigma-Aldrich, USA), previously equilibrated with a buffer containing 15% n-propyl alcohol and 0.05 M sodium acetate (pH=4.7). The column was eluted with an increasing n-propyl alcohol gradient for LTA purification. The eluted LTA was applied on a Q-sepharose ion exchange column and refined by increasing the concentration of NaCl, followed by the vacuum evaporation.

For PGN purification, heat-treated L. paracasei GMNL®-143 was washed 3 times with sterile PBS, lyzed with an ultrasonic homogenizer, and centrifuged at 8000 rpm for 3 minutes at 4°C. The pellets were dissolved in sterile distilled water and centrifuged again. This step above was repeated 3 times and the precipitates were treated with 100 mg/mL RNase and 50 mg/mL DNase for 18 hours, followed by another 18-hour incubation with 200 mg/mL trypsin at 37°C. After centrifugation

(8000 rpm, 30 minutes, 4°C), the final precipitates which were bacterial cell walls were freeze-dried, redissolved in 5% trichloroacetic acid (TCA) and incubated for 18 hours at 22°C, centrifuged again (8000 rpm, 30 minutes), rinsed 3 times with sterile distilled water and acetone respectively, and then dried to produce PGN powders.

Biofilm formation assay

0.1 mL of 2×10^8 cells/mL *S. mutans* ATCC 25175 suspension and 0.1 mL of LTA or PGN extracted from *L. paracasei* GMNL®-143 were added into a 96-well cell culture plate, and co-incubated at 37°C for 24 hours to form biofilms. The plates were carefully washed 3 times with PBS to remove unbound and weakly adsorbed bacteria. The biofilms were fixed with 95% ethanol for 1 minute, stained with 0.1% crystal violet for 15 minutes, washed carefully at least 3 times with PBS, and dried in a laminar flow hood. 180 uL of glacial acetic acid was added into wells to dissolve the crystal violet in bacteria and the absorbance at 595 nm of the wells was measured. High absorbance value represented a high biofilm formation.

II. Supporting Tables

Table S1. Genus-specific and species-specific primers for quantitative real-time PCR.

Specificity	Sequence (from 5' to 3')	PCR product
All bacteria		150 bp
Forward	GTGSTGCAYGGYTGTCGTCA	
Reverse	ACGTCRTCCMCACCTTCCTC	
Porphyromonas		168 bp
Forward	CCATAACGAGCGCAACCCAC	
Reverse	GGTAGCTGCCCATTGTCCCT	
Treponema		140 bp
Forward	GAGGAAGGCCGGAAGGTTGT	
Reverse	GCTCGCGCCTTACGTGTTAC	
Tannerella		116 bp
Forward	AGACGACGGAGAGTGAGAGC	
Reverse	CAGGGTTGCGCTCGTTATGG	
Filifactor		116 bp
Forward	GAGAGGGTGAACGGCCACAT	
Reverse	CGTTGCTGCATCAGGGTTCC	
Aggregatibacter		150 bp
Forward	ACGCGAAGAACCTTACCTACTCT	
Reverse	ATAAGGGTTGCGCTCGTTGC	
Streptococus mutans		136 bp
Forward	CGCGTAGGTAACCTGCCTAT	
Reverse	GCTAATACAACGCAGGTCCA	
Porphyromonas gingivalis		119 bp
Forward	ATAGGCATGCGTCCCATTAG	
Reverse	CGTAGGAGTCTGGTCCGTGT	
Fusobacterium nucleatum		101 bp
Forward	CGCAGAAGGTGAAAGTCCTGTAT	
Reverse	TGGTCCTCACTGATTCACACAGA	
Aggregatibacter actinomycetemcomitans		77 bp
Forward	CTTACCTACTCTTGACATCCGAA	
Reverse	ATGCAGCACCTGTCTCAAAGC	
Streptococcus		190 bp
Forward	TGCACCACCTGTCACCTCTG	
Reverse	CGCATTAAGCACTCCGCCTG	
Haemophilus		105 bp
Forward	TGGCGGACGGGTGAGTAATG	
Reverse	GGTCCCGCACTTTCGTCTCT	
Capnocytophaga		199 bp
Forward	AGGGTCTGTAGGCGGGCTAT	
Reverse	ACGCTTTCGTCTCTCAGCGT	
Gemella		115 bp
Forward	GTCGCGGTGAATACGTTCCC	
Reverse	CTATCCCACCGTGACCGGC	
Campylobacter		123 bp
Forward	GGCTTACCAAGGCTATGACGC	
Reverse	AGGCTTTCGCCCATTGAGCA	
Granulicatella		167 bp
Forward	GAGCGCAGGCGGTCAATTA	
Reverse	ACCAGAGAGTCGCCTTCGC	

Table S2. Screening co-aggregation oral pathogens abilities of many strains from 8 Lactobacillus species.

Probiotic		co-aggregation ability with oral pathogen[a]			
Species	strain	SM	PG	FN	PI
L. paracasei	GMNL-39	+	+	+	−
	GMNL-43	+	+	+	−
	GMNL-143	++	++	++	+
	BCRC-16100	−	−	++	−
	GMNL-855	−	−	++	−
L. salivarius	GMNL-72	−	−	++	−
	GMNL-212	−	++	++	−
	GMNL-214	−	−	++	−
	GMNL-215	−	+	++	−
L. reuteri	GMNL-89	−	−	+	−
	GMNL-101	−	−	+	−
	GMNL-340	−	−	+	−
	GMNL-348	−	−	+	−
L. fermentum	GMNL-67	−	+	+	−
	GMNL-69	−	+	+	−
	GMNL-75	−	+	+	−
L. helveticus	GMNL-54	−	−	+	+
	GMNL-591	−	+	+	++
	GMNL-597	−	+	+	++
L. gasseri	GMNL-83	−	+	+	+
	GMNL-105	−	−	+	+
	GMNL-416	−	+	+	+
	GMNL-497	−	+	+	++
L. plantarum	GMNL-1	−	+	−	++
	GMNL-2	−	+	−	++
	GMNL-3	−	+	−	++
	GMNL-4	−	+	−	++
L. acidophilus	GMNL-245	−	+	++	++
	GMNL-332	+	+	+	++
	GMNL-423	−	+	+	+

[a]Oral pathogens includes *S. mutans* (SM), *P. gingivalis* (PG), F. nucleatum (FN), or *P. intermedia* (PI). The co-aggregation ability was represented as: −, no co-aggregation

or co-aggregation starting time >30 min; +, co-aggregation starting time about 3-30 min; ++, co-aggregation starting time about 3-30 min <3 min.

Table S3. General genomic features of *Lactobacillus paracasei* GMNL®-143.

	GMNL®-143
Size (bp)	3,000,366
G+C content (%)	46.33
Total genes	2,867
Coding content (%)	84.94
Gene average length (bp)	873
Genes assigned to COGs[a]	2,404 (83.9%)
Chromosome	1
rRNA operons	5
tRNA	62
plasmids	1
Transposases	70
CRISPR loci	0
Prophage-like clusters	1
Bacteriocin	3

[a]COGs, Cluster of Orthologous Groups

Table S4. Unique genes of *L. paracasei* GMNL®-143, GMNL®-855 and BCRC 16100.

COG class	Description	Gene name		
		GMNL®-143	GMNL®-855	BCRC 16100
E	Amino acid transport and metabolism	cjaA	cysE, mccA	-
G	Carbohydrate transport and metabolism	-	xylP	pts32BC, gatY, mnaA
M	Cell wall/membrane/envelope biogenesis	capM, epsG	rfbP, rgpAc, mprF	kdsD, tuaG, tagF, ywqC, tagE, gtf1
L	Secondary metabolites biosynthesis, transport, and catabolism	traA	cas2, cas1, cas9, tnpR, yqaJ, recT, rusA, pi112	-
U	Intracellular trafficking, secretion, and vesicular transport	-	clpP	secY2, secA2
E	Amino acid transport and metabolism	-	clpP	secY2, secA2
P	Inorganic ion transport and metabolism	glnP7, glnP9	-	kdgT, sfuB, fbpC

Table S5. Minimum inhibitory concentrations (MICs) of *L. paracasei* GMNL-143 towards 8 antimicrobials and the microbiological cut-off values.

Antbiotics	EFSA cut-off (g/L)	GMNL-143 MIC (g/L)
Ampicillin	4	2
Gentamicin	32	8
Kanamycin	64	64
Streptomycin	64	32
Erythromycin	1	0.25
Clindamycin	1	0.125
Tetracycline	4	2
Chloramphenicol	4	4

Table S6. Inhibitory ability of *L. paracasei* GMNL®-143 to oral pathogens by inhibition zone assay.

	Inhibition ability		
	S. mutant	*P. gingivalis*	*F. nucleatum*
GMNL®-143 supernatant	−	+	++
GMNL®-143 supernatant (heat-treated)	−	+	++
GMNL®-143 supernatant (neutralized to pH=6)	−	−	−
Heat-killed GMNL®-143 powder	−	−	−

Table S7. Bacterial composition of plaques from gingivitis patients according to the experimental serials[a].

	Serial 1, GMNL®-143 → Placebo, N =10			Serial 2, Placebo → GMNL®-143, N =7		
	Placebo	GMNL®-143	*p*-value[b]	Placebo	GMNL®-143	*p*-value[b]
$2^{-\Delta\Delta CT}$	Mean ± SD	Mean ± SD		Mean ± SD	Mean ± SD	
Total bacteria	0.92 ± 0.65	0.80 ± 0.29	0.8457	0.98 ± 0.33	0.99 ± 0.40	0.8125
Porphyromonas	10.18 ± 17.24	4.60 ± 11.75	0.1049	0.87 ± 0.83	1.81 ± 1.91	0.5350
Treponema	1.72 ± 2.17	2.64 ± 2.06	0.2110	0.91 ± 0.50	1.88 ± 1.45	0.1649
Tannerella	4.86 ± 6.11	0.59 ± 0.32	0.0164*	0.90 ± 0.83	1.73 ± 1.03	0.1282
Filifactor	1.07 ± 0.45	1.38 ± 1.11	0.7394	1.37 ± 0.44	1.35 ± 0.65	0.7104
Aggregatibacter	3.33 ± 4.60	0.82 ± 1.08	0.0535	0.69 ± 0.35	1.32 ± 1.14	0.4557
Streptococus mutans	0.77 ± 1.04	1.90 ± 2.65	0.1128	2.30 ± 2.81	2.05 ± 1.61	0.5887
Porphyromonas gingivalis	1.45 ± 1.55	0.83 ± 0.72	0.4359	0.89 ± 1.06	1.00 ± 1.00	0.8357
Fusobacterium nucleatum	0.79 ± 0.24	1.25 ± 0.62	0.0653	1.21 ± 0.78	1.16 ± 0.60	0.9015
Aggregatibacter actinomycetemcomitans	1.94 ± 1.73	0.77 ± 1.06	0.1613	1.23 ± 0.90	2.19 ± 2.96	0.9015
Streptococcus	1.03 ± 0.84	0.60 ± 0.28	0.4002	1.64 ± 0.87	1.25 ± 1.09	0.3829
Haemophilus	2.16 ± 2.49	1.63 ± 2.58	0.3527	0.83 ± 0.47	1.56 ± 1.10	0.1649
Capnocytophaga	1.71 ± 1.81	1.44 ± 1.54	0.7304	1.33 ± 1.16	1.58 ± 1.38	0.7308
Gemella	1.05 ± 0.80	0.82 ± 0.79	0.4470	1.64 ± 1.33	1.33 ± 0.56	0.9015
Campylobacter	1.78 ± 2.21	3.15 ± 3.10	0.2428	0.67 ± 0.39	1.35 ± 1.09	0.3829
Granulicatella	0.65 ± 0.69	0.66 ± 0.68	>0.9999	0.77 ± 0.79	1.32 ± 1.11	0.6282

[a]Bacterial composition was determined by the qPCR method and calculating the value of $2^{-\Delta\Delta Ct}$ that reflected the fold change compared to the baseline of bacterial contents from plaque or GCF specimens.

[b]Statistical analyses between GMNL®-143 and placebo toothpaste were performed by Wilcoxon signed-rank test. *, $p< 0.05$.

Table S8. Bacterial composition of gingival crevicular fluid from gingivitis patients according to the experimental serials[a].

	Serial 1, GMNL®-143 → Placebo, N =10			Serial 2, Placebo → GMNL®-143, N =7		
	Placebo	GMNL®-143	*p*-value[b]	Placebo	GMNL®-143	*p*-value[b]
$2^{-\Delta\Delta CT}$	Mean ± SD	Mean ± SD		Mean ± SD	Mean ± SD	
Total bacteria	0.97 ± 0.81	0.92 ± 0.92	>0.9999	2.20 ± 2.27	1.31 ± 0.70	0.5781
Porphyromonas	2.45 ± 2.82	2.27 ± 2.43	0.8633	0.91 ± 1.21	1.07 ± 0.44	0.3176
Treponema	2.16 ± 2.75	1.68 ± 1.89	0.9626	1.14 ± 0.73	4.49 ± 4.61	0.0175*
Tannerella	2.17 ± 1.95	1.58 ± 1.53	0.4082	0.74 ± 0.46	1.88 ± 1.46	0.0732
Filifactor	2.09 ± 1.63	1.57 ± 1.14	0. 4813	1.22 ± 0.52	1.45 ± 0.81	0.9015
Aggregatibacter	2.42 ± 3.37	1.60 ± 1.47	>0.9999	0.96 ± 0.74	1.43 ± 0.73	0.2593
Streptococcus mutans	1.02 ± 0.78	0.48 ± 0.31	0.0625	2.17 ± 2.56	0.81 ± 0.94	0.3660
Porphyromonas gingivalis	0.86 ± 0.48	1.07 ± 0.66	0.5787	0.32 ± 0.19	0.97 ± 1.00	0.5350
Streptococcus	0.85 ± 0.38	0.69 ± 0.30	0.3150	1.40 ± 1.06	1.12 ± 0.90	0.5350
Haemophilus	0.99 ± 1.21	0.89 ± 0.81	0.7802	1.75 ± 1.74	1.95 ± 1.16	0.7104
Capnocytophaga	2.24 ± 2.55	2.08 ± 1.46	0.7394	0.79 ± 0.50	1.36 ± 1.58	>0.9999
Gemella	0.48 ± 0.29	0.37 ± 0.31	0.3527	0.92 ± 0.78	1.78 ± 1.66	0.3095
Campylobacter	1.51 ± 1.38	2.48 ± 2.06	0.4967	1.33 ± 1.19	2.27 ± 2.29	0.6200
Granulicatella	0.69 ± 0.50	0.97 ± 1.30	0.7394	0.71 ± 0.88	1.91 ± 2.77	0.4557

[a]Bacterial composition was determined by the qPCR method and calculating the value of $2^{-\Delta\Delta Ct}$ that reflected the fold change compared to the baseline of bacterial contents from plaque or GCF specimens.

[b]Statistical analyses between GMNL®-143 and placebo toothpaste were performed by Wilcoxon signed-rank test. *, $p < 0.05$.

III. Supporting figures

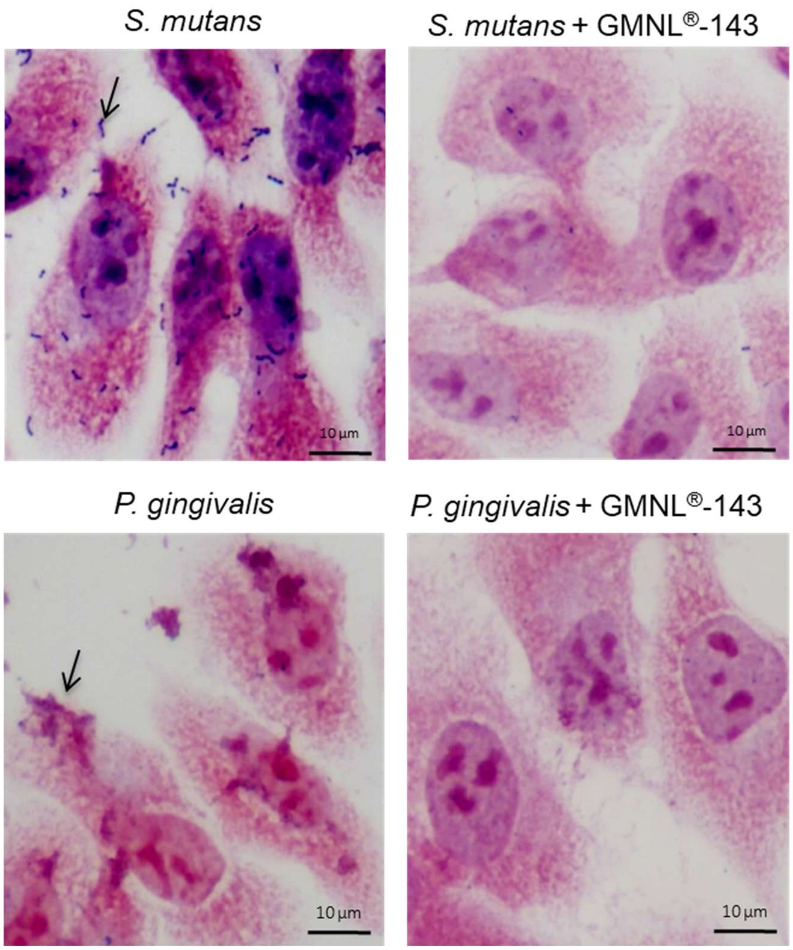

Figure S1. Co-aggregation of GMNL®-143 decreased the adhesion of oral pathogens to SG cells. SG human gingival epithelial cells were co-incubated with the upper layer bacterial solution from *L. paracasei* GMNL®-143 with or without oral bacterial pathogens in co-coaggregation assay. Cells were then stained by Gram staining and were photographed under a light microscope. Scale bars, 10 µm.

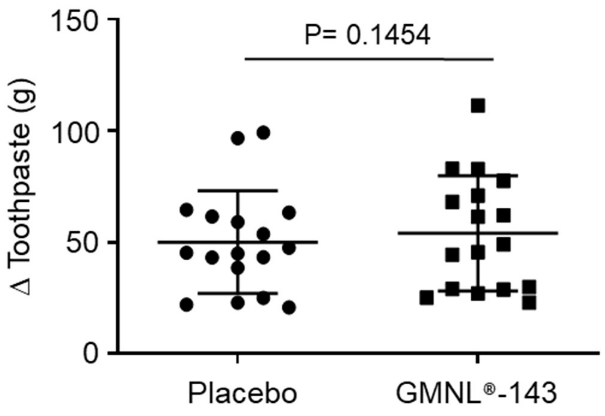

Fig. S2. The average toothpaste usage between groups. The toothpaste tubes of both groups were collected and weighed after use. Student's t-test was used for statistical analysis.

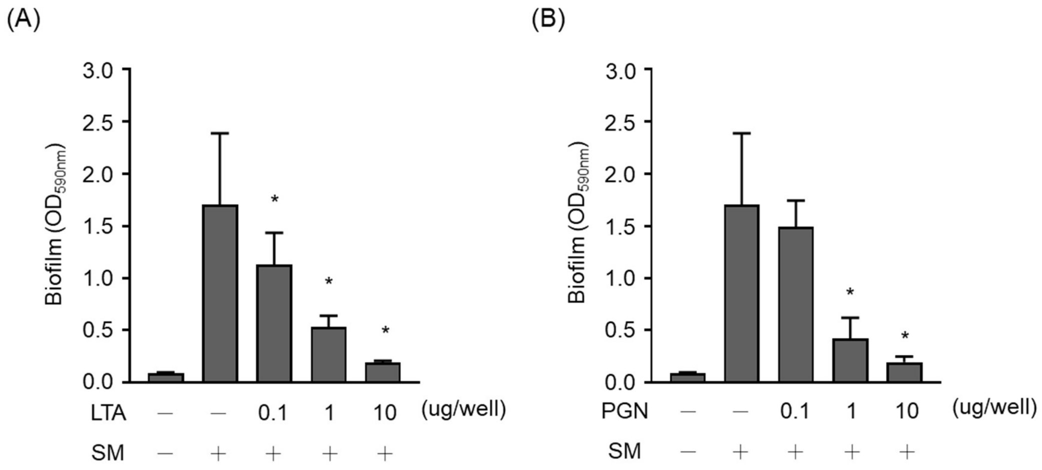

Fig. S3. Lipoteichoic acid and Peptidoglycan from GMNL®-143 inhibited S. mutans biofilm formation. Different concentrations of Lipoteichoic acid (LTA) (A) and Peptidoglycan (PGN) (B) that extracted from *L. paracasei* GMNL®-143 were used for co-culturing with *S. mutans* in wells of a 96-well cell culture plate. Biofilms formations by *S. mutans* were stained with crystal violet. The stained biofilms were then dissolved with glacial acetic acid and the absorbances at 595 nm were measured.

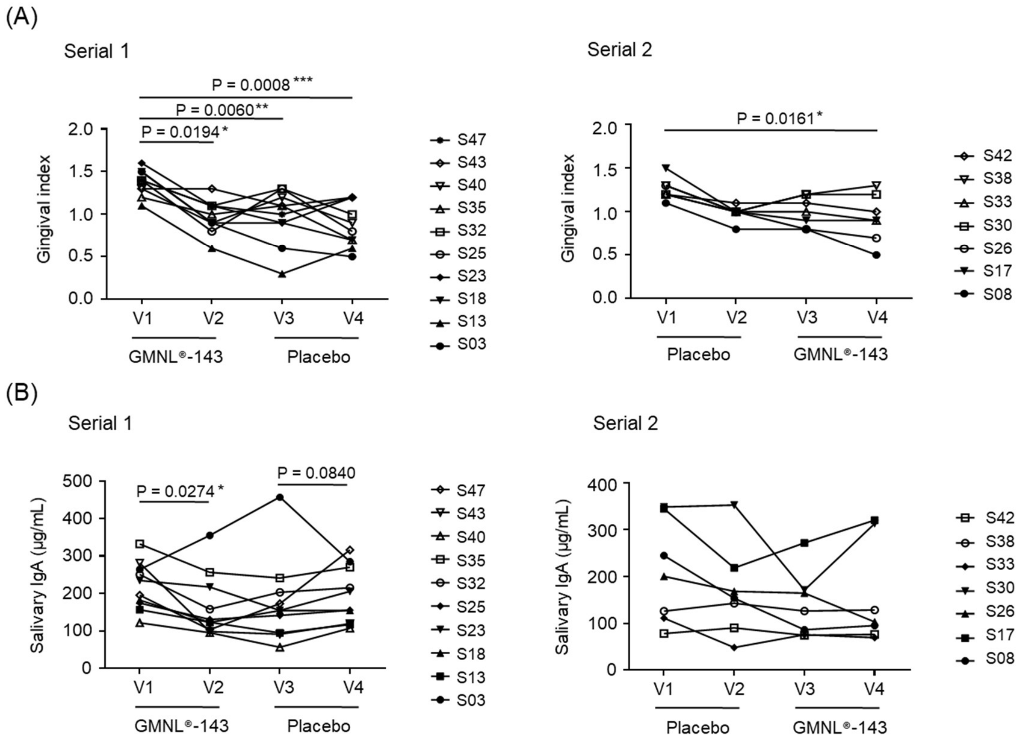

Figure S4. GMNL®-143 toothpastes improve the clinical parameters of gingivitis, especially in a GMNL®-143 → Placebo treatment sequence. Gingivitis index (A) and salivary IgA (B) were plotted based on the measurements taken at each visit of serial 1 (GMNL®-143 → Placebo) and serial 2 (Placebo → GMNL®-143) groups. Dunn's multiple comparison test was used for statistical analysis. *, $p < 0.05$.
